# Prevalence and correlates of depressive symptoms among Ethiopian and Ethiopian-Descent adults in the Seattle metropolitan area: A cross-sectional study

**DOI:** 10.1371/journal.pmen.0000736

**Published:** 2026-10-21

**Authors:** Hiwot Weldemariam, Yonathan Dagnew, Meron Girma, Mehari Worku, Daniel A. Enquobahrie

**Affiliations:** 1 Department of Epidemiology, University of Washington, Seattle, Washington, United States of America; 2 Center for Firearm Injury Prevention, University of Washington, Seattle, Washington, United States of America; 3 Department of Psychology, University of Washington, Seattle, Washington, United States of America; 4 Middle Eastern Language and Culture, University of Washington, Seattle, Washington, United States of America; PLOS: Public Library of Science, UNITED KINGDOM OF GREAT BRITAIN AND NORTHERN IRELAND

## Abstract

Depression is a common and disabling condition, yet population-level estimates among Ethiopian and Ethiopian-descendant adults in the Seattle metropolitan area are limited. We conducted a cross-sectional community-based survey from November to December 2024 among adults aged ≥18 years who self-identified as Ethiopian or Ethiopian-descendant. Participants were recruited through social media, community networks, and faith-based organizations and completed anonymous REDCap questionnaires. Clinically significant depressive symptoms were defined as a Patient Health Questionnaire-9 (PHQ-9) score ≥10, a screening threshold rather than a clinical diagnosis. We used logistic regression to examine associations between clinically significant depressive symptoms and the primary exposures of nativity, self-rated physical health, chronic medical conditions, reproductive health history, recreational marijuana or other drug use, and firearm exposure, with adjustment for prespecified sociodemographic covariates. A comprehensive model included the primary full-sample exposures simultaneously, with Benjamini-Hochberg correction for multiple comparisons. Among 474 participants (mean age, 31.6 years; 49.8% male), 178 (37.6%) screened positive for clinically significant depressive symptoms. In the mutually adjusted model, poorer self-rated physical health was associated with higher odds of depressive symptoms: average health (adjusted odds ratio [aOR], 3.83; 95% CI, 2.08–7.36) and poor/somewhat poor health (aOR, 10.73; 95% CI, 4.53–26.80), compared with excellent health. Ever use of recreational marijuana or other drugs (aOR, 3.52; 95% CI, 2.08–6.03) and knowing someone injured or killed by a firearm in the past year (aOR, 2.32; 95% CI, 1.39–3.90) were also associated with higher odds. Among parous women, a history of postpartum depression was associated with current depressive symptoms (aOR, 9.76; 95% CI, 4.31–23.51). Clinically significant depressive symptoms were common in this sample and were associated with poorer physical health, recreational marijuana or other drug use, firearm-related exposure, and prior postpartum depression. Longitudinal research is needed to clarify temporal relationships and inform culturally responsive prevention and care.

## Introduction

Epidemiological studies suggest that nearly half of U.S. adults will meet diagnostic criteria for at least one mental disorder by age 75, underscoring the substantial public health burden of psychiatric conditions [1]. Depression is among the most common and disabling mental health conditions in the United States [2]. In 2024, an estimated 19% of U.S. adults reported ever having been told by a healthcare professional that they had a depressive disorder, while approximately 5% reported experiencing depressive symptoms regularly [3]. Despite the availability of effective treatments, substantial disparities persist in the burden, recognition, and treatment of depressive disorders, particularly among racial and ethnic minority and immigrant populations [4–7].

Immigrants comprised approximately 14% of the U.S. population in 2022, with a growing proportion originating from sub-Saharan Africa [8–10]. The African-born population in the United States has increased substantially since 2000 and now constitutes a large share of the foreign-born Black population [11]. Mental health among immigrants and refugees is shaped by experiences occurring before, during, and after migration [9]. Individuals from conflict-affected regions may experience war, forced displacement, gender-based violence, family separation, and other traumatic events before migration [10,12–14]. After resettlement, additional stressors may include employment insecurity, language barriers, discrimination, acculturation pressures, social isolation, and limited access to culturally responsive mental health services [15,16]. Although some immigrant groups initially demonstrate a “healthy immigrant effect,” this advantage may diminish with longer duration of residence or across generations as social and structural stressors accumulate [9]. Mental health patterns among immigrant populations are heterogeneous, however, and vary by migration history, country of origin, resettlement context, and social conditions [17,18].

The Seattle metropolitan area is home to a growing African immigrant population, including a substantial Ethiopian community [19]. Yet East African immigrants remain underrepresented in mental health research and are frequently grouped into broad racial categories such as “Black” or “African American,” which may obscure differences in migration history, culture, social context, and healthcare experiences. Evidence specific to Ethiopian and Ethiopian-descendant adults in the Seattle metropolitan area remains limited. Prior research suggests that stigma, limited mental health literacy, fear of social judgment, mistrust of healthcare systems, language barriers, and limited availability of culturally responsive services may reduce help-seeking within African immigrant communities [20]. These barriers may contribute to unmet mental health needs and make it difficult to characterize depressive symptom burden in this population.

This study aimed to estimate the prevalence of clinically significant depressive symptoms and examine their associations with nativity, self-rated physical health, chronic medical conditions, reproductive health history, recreational marijuana or other drug use, and firearm-related exposure among Ethiopian and Ethiopian-descendant adults in the Seattle metropolitan area. By focusing on an understudied community, this study seeks to provide preliminary evidence that may inform culturally responsive mental health outreach, community-based support, and future longitudinal and population-based research.

## Materials and methods

### Ethics statement

Online informed consent was obtained via REDCap before participants began the screening survey. A waiver of signature was granted to protect participants’ confidentiality, and the consent form explicitly stated that the study was anonymous. The study protocol was reviewed by the University of Washington Institutional Review Board and determined to meet criteria for exempt status

### Study setting and population

A cross-sectional community-based survey was conducted from November through December 2024 among adults of Ethiopian or Ethiopian-descendant background residing in the Seattle metropolitan area. For this study, the Seattle metropolitan area was defined as the Seattle–Tacoma–Bellevue region, including King, Pierce, and Snohomish counties, consistent with the U.S. Census Bureau definition [21]. Participants were recruited using nonprobability convenience sampling through social media platforms (Instagram, Facebook, WhatsApp, and Viber), outreach through the study team’s community networks, and partnerships with local churches and community-based organizations. Eligibility criteria included being aged 18 years or older, self-identifying as Ethiopian or Ethiopian-descendant, and residing in the study area. The survey was administered in English. A total of 474 eligible participants were included in the analytic sample.

The study protocol was reviewed by the University of Washington Institutional Review Board and determined to meet criteria for exempt status. Participants provided electronic informed consent through REDCap before beginning the survey. A waiver of written signature was granted to protect confidentiality, and participants were informed that survey responses were anonymous.

#### Data collection and variable specification.

We utilized REDCap, a secure and HIPAA-compliant web-based survey and data management platform, to administer the survey [22]. Participants accessed a brief screening questionnaire through the platform to confirm their eligibility. Only those who met the eligibility criteria were prompted to continue to complete the full survey; which took approximately 20 minutes to complete. After each submission, a study team member reviewed the data for authenticity and duplication by following recommended practices for online research [23]. Participants who completed the survey were then provided a $10 electronic gift card. To maintain survey anonymity, participants were redirected to a separate link after completing the survey to provide contact information for gift card distribution.

### Survey instrument

The survey consisted of 48 questions organized into four sections: (1) sociodemographic characteristics, (2) physical and reproductive health, (3) mental and behavioral health, and (4) firearm-related experiences and perceptions. Sociodemographic measures included age, sex, educational attainment, employment status, marital status, nativity, and duration of residence in the United States. Physical and reproductive health measures included self-rated physical health, self-reported chronic medical conditions, history of childbirth, number of live births, and history of postpartum depression. Depressive symptoms were assessed using the nine items of the Patient Health Questionnaire-9 (PHQ-9). Additional mental health items assessed self-rated mental health, prior mental health diagnosis, use of mental health services, and perceived barriers to care. Behavioral health items assessed recreational marijuana or other drug use. The firearm-related section assessed experiences and perceptions related to firearm violence, including two separate items on interpersonal firearm exposure: whether participants had personally known someone who was injured or killed by a firearm in their community during the past year, and the number of people they knew who had experienced firearm injury or death. The section also assessed perceptions of firearm violence in the community and concerns about becoming a victim of firearm violence.

### Outcome: Depressive symptoms

The main outcome of interest, depressive symptoms, were assessed using items from the PHQ-9, a widely validated screening instrument used in both clinical and community research settings [24]. The PHQ-9 assesses the frequency of nine depressive symptoms during the preceding two weeks. Each item is scored from 0 (“Not at all”) to 3 (“Nearly every day”), yielding a total score ranging from 0 to 27. The primary outcome was clinically significant depressive symptoms, defined as a PHQ-9 score ≥10. This cutoff was used as a screening threshold and was not interpreted as a clinical diagnosis of depression [25]. Item 9 of the PHQ-9 assesses thoughts of self-harm. Because the survey was anonymous, individual responses could not be linked back to participants for direct clinical follow-up. All participants were therefore provided with mental health and crisis resources, including crisis hotline information and local support services, and were encouraged to seek immediate assistance if experiencing distress or thoughts of self-harm. Survey anonymity was maintained to protect confidentiality and support disclosure of sensitive experiences

### Exposures

The primary exposures of interest included nativity, self-rated physical health, chronic medical conditions, reproductive health history, behavioral factors, and firearm-related exposure. Nativity was assessed using two measures: foreign-born status (yes/no) and, among foreign-born participants, duration of residence in the United States categorized as 2–5, 6–10, 11–20, and >20 years. Self-rated physical health was assessed using the response options Excellent, Average, Somewhat poor, Poor, and Not sure. For regression analyses, Somewhat Poor and Poor were combined into a single category, with Excellent as the reference group; Not sure responses were excluded because they did not represent an ordered health-status category. Chronic medical conditions included self-reported diabetes, hypertension, cancer, chronic respiratory disease, kidney disease, and stroke. For primary regression analyses, these conditions were combined into a binary indicator of any listed chronic medical condition (yes/no). Individual conditions were described in Table 1 but were not modeled separately. Reproductive health variables included history of childbirth, parity, and history of postpartum depression. Analyses of childbirth were restricted to female participants, while parity and postpartum depression analyses were restricted to parous women. For postpartum depression, participants who responded “Not sure” were excluded from the primary regression comparison. The primary behavioral exposure was self-reported ever use of recreational marijuana or other drugs (yes/no). Firearm-related exposure was defined as personally knowing someone who had been injured or killed by a firearm in the participant’s community during the past year (yes/no).

**Table 1:** Characteristics of Ethiopian and Ethiopian-descendant adults in the Seattle metropolitan area (N = 474).

Characteristic	Overall, n (%)*
**Age, years, mean (SD)**	31.6 (6.5)
**Age category**	
18–25	82 (17.3)
26–30	167 (35.3)
31–35	127 (26.7)
36–40	62 (13.1)
> 40	36 (7.6)
**Sex**	
Male	236 (49.8)
Female	238 (50.2)
**Education**	
High school or less	79 (16.7)
College degree	273 (57.6)
Postgraduate education	122 (25.7)
**Employment status**	
Employed full-time	313 (66.0)
Employed part-time	109 (23.0)
Student	14 (3.0)
Self-employed	22 (4.6)
Unemployed	13 (2.7)
Retired	3 (0.6)
**Marital status**	
Single	172 (36.3)
Married	273 (57.6)
Separated	20 (4.2)
Divorced	6 (1.3)
Widowed	3 (0.6)
**Born outside the United States**	
No	215 (45.4)
Yes	254 (53.6)
Missing	5 (1.1)
**Years in the United States among foreign-born participants (N = 240)**	
2–5 years	15 (6.2)
6–10 years	80 (33.3)
11–20 years	107 (44.6)
> 20 years	38 (15.8)
**Self-rated physical health**	
Excellent	142 (30.0)
Average	266 (56.1)
Somewhat poor	38 (8.0)
Poor	22 (4.6)
Not sure	6 (1.3)
**Self-rated mental health**	
Excellent	112 (23.6)
Average	253 (53.4)
Somewhat poor	72 (15.2)
Poor	33 (7.0)
Not sure	4 (0.8)
**Any of the listed chronic medical conditions**	
No	340 (71.7)
Yes	134 (28.3)
**Specific chronic medical conditions among participants with at least one condition (N = 134)†**	
Hypertension	68 (50.7)
Diabetes	43 (32.1)
Chronic respiratory disease	31 (23.1)
Kidney disease	7 (5.2)
Cancer	7 (5.2)
Stroke	3 (2.2)
**Smoking frequency**	
Never	206 (43.5)
More than once every day	43 (9.1)
Once every day	86 (18.1)
Once a week	139 (29.3)
**Vaping frequency**	
Never	295 (62.2)
More than once every day	20 (4.2)
Once every day	57 (12.0)
Once a week	102 (21.5)
**Alcohol use frequency**	
Never	98 (20.7)
More than once every day	37 (7.8)
Once every day	88 (18.6)
Once a week	251 (53.0)
**Ever used recreational marijuana or other drugs**	
No	336 (70.9)
Yes	138 (29.1)
**Prior mental health diagnosis**	
No	393 (82.9)
Not sure	5 (1.1)
Yes	76 (16.0)
**Last mental health examination**	
< 6 months ago	91 (19.2)
6 months to 1 year ago	196 (41.4)
> 1 year ago	81 (17.1)
Never	106 (22.4)
**Ever seen a therapist**	
No	250 (52.7)
Yes	224 (47.3)
**Barriers to seeking mental health care†**	
Stigma	228 (48.1)
Lack of awareness	218 (46.0)
Cost	255 (53.8)
Limited culturally appropriate services	170 (35.9)
Transportation problems	104 (21.9)
**Ever given birth, female participants only (N = 238)**	
No	55 (23.1)
Yes	183 (76.9)
**Number of live births, parous women only (N = 183)**	
1	88 (48.1)
2	77 (42.1)
≥ 3	16 (8.7)
Missing	2 (1.1)
**History of postpartum depression, parous women only (N = 183)**	
No	110 (60.1)
Not sure	3 (1.6)
Yes	70 (38.3)
**Pregnancy-related complications, parous women only (N = 183)†**	
Cesarean section delivery	67 (36.7)
Gestational diabetes	14 (7.6)
Hypertension	21 (11.5)
Low birth weight	23 (12.6)
Preterm labor	14 (7.7)
None	74 (40.4)
**Believes firearm violence is an issue in the community**	
No	132 (27.8)
Not sure	20 (4.2)
Yes	322 (67.9)
**Worried about being a victim of firearm violence**	
Not at all worried	34 (7.2)
Not too worried	65 (13.7)
Somewhat worried	217 (45.8)
Very worried	158 (33.3)
**Personally knew someone injured or killed by a firearm** **in the community during the past year**	
Yes	251(53.0)
No	223(47.0)
**Number of people known who were injured or killed by a firearm in the past year**	
None	208 (43.9)
1–3	190 (40.1)
> 3	76 (16.0)

* Values are n (%) unless otherwise indicated.

† Multiple responses were permitted; therefore, percentages do not sum to 100%. Among 183 parous women, parity information was available for 181 participants; percentages for number of live births are based on those 181 participants. The two firearm-related measures were separate survey items: one assessed the number of people known to have experienced firearm injury or death, and the other assessed whether participants had personally known someone injured or killed by a firearm in their community during the past year.

### Covariates

Sociodemographic characteristics were selected a priori as adjustment covariates based on prior literature and their theoretical relevance to depressive symptoms [26–29]. Age was modeled continuously in years. Sex was categorized as male or female. Marital status was dichotomized as married versus not married for regression analyses because of small cell sizes in several categories; all observed marital status categories are presented descriptively in Table 1. Educational attainment was categorized as high school or less, college degree, or postgraduate education. Employment status was categorized as employed full-time, employed part-time, student, self-employed, unemployed, or retired. Exposure-specific adjusted models included age, sex, marital status, educational attainment, employment status, and nativity, except when nativity or years in the United States was the exposure of interest.

### Data analysis

Descriptive statistics were used to summarize participant characteristics. Continuous variables were summarized using means and standard deviations, and categorical variables using frequencies and percentages. The primary outcome was clinically significant depressive symptoms, defined as a PHQ-9 score ≥10; this threshold was treated as a screening indicator rather than a clinical diagnosis.

Crude associations between each exposure and clinically significant depressive symptoms were evaluated using separate logistic regression models. Exposure-specific adjusted models included age, sex, marital status, educational attainment, employment status, and nativity, except when nativity or duration of residence in the United States was the exposure of interest. Crude and adjusted odds ratios (ORs and aORs) with 95% confidence intervals (CIs) were reported.

To assess whether associations persisted after accounting for correlation among exposures, we additionally fit a comprehensive multivariable logistic regression model that simultaneously included self-rated physical health, any chronic medical condition, ever use of recreational marijuana or other drugs, firearm exposure in the past year, and nativity, together with the prespecified sociodemographic covariates. Duration of residence in the United States was analyzed separately among foreign-born participants because it was not defined for U.S.-born participants. Reproductive health analyses were conducted separately. History of childbirth was evaluated among female participants, whereas number of live births and history of postpartum depression were evaluated among parous women. Participants responding “Not sure” to postpartum depression were excluded from the primary Yes-versus-No comparison. Estimates for parity were interpreted cautiously because of limited precision in some categories.

Benjamini-Hochberg false discovery rate correction was applied separately within prespecified families of adjusted exposure comparisons. The primary exposure-specific analyses included 9 comparisons, the reproductive-health analyses included 4 comparisons, and the comprehensive logistic regression model included 6 exposure coefficients. Correction was therefore performed within analytic families rather than globally across all statistical tests; this approach is less conservative than a single global multiple-comparison adjustment. The parity trend test was evaluated separately and was not included in the reproductive-health correction family.

As a sensitivity analysis, the PHQ-9 score was modeled continuously using linear regression. Both exposure-specific adjusted models and a comprehensive mutually adjusted linear model were fit using the same exposure and covariate structure as the primary analyses. Benjamini-Hochberg correction was applied separately to the corresponding sensitivity-analysis families, comprising 9 exposure-specific comparisons and 6 exposure coefficients in the comprehensive continuous model. Missing data were handled using complete-case analysis; therefore, analytic sample sizes varied across models. All statistical tests were two-sided, with statistical significance defined as p < 0.05. Analyses were conducted using R version 4.4.1.

## Results

Among 474 Ethiopian and Ethiopian-descendant adults, the mean age was 31.6 years (SD, 6.5), and 50.2% were female (Table 1). Most participants were married (57.6%) or single (36.3%). Educational attainment was high, with 57.6% reporting a college degree and 25.7% reporting postgraduate education. Overall, 66.0% were employed full-time and 23.0% were employed part-time.

Among participants with available nativity data, 54.2% were born outside the United States. Among foreign-born participants with available duration-of-residence data (N = 240), 44.6% had lived in the United States for 11–20 years, 33.3% for 6–10 years, 15.8% for more than 20 years, and 6.2% for 2–5 years.

Most participants rated their physical health as average (56.1%) or excellent (30.0%), while 8.0% rated their health as somewhat poor and 4.6% as poor. At least one of the listed chronic medical conditions was reported by 28.3% of participants. Among those with at least one chronic condition, the most commonly reported conditions were hypertension (50.7%), diabetes (32.1%), and chronic respiratory disease (23.1%).

Among female participants (N = 238), 183 (76.9%) reported having given birth. Among parous women with available parity data (N = 181), 48.6% reported one live birth, 42.5% reported two, and 8.8% reported three or more. Pregnancy-related complications included cesarean delivery (36.7%), low birth weight (12.6%), hypertension (11.5%), preterm labor (7.7%), and gestational diabetes (7.6%). Among parous women, 38.3% reported a history of postpartum depression, while 1.6% responded “Not sure.”

More than half of participants rated their mental health as average (53.4%), while 23.6% rated it as excellent, 15.2% as somewhat poor, and 7.0% as poor. A prior mental health diagnosis was reported by 16.0% of participants, and 47.3% had ever seen a therapist. Participants identified several barriers to seeking mental health care, most commonly cost (53.8%), stigma (48.1%), lack of awareness of available services (46.0%), limited culturally appropriate services (35.9%), and transportation problems (21.9%).

Ever use of recreational marijuana or other drug use was reported by 29.1% of participants. Regarding other substance-use behaviors, 43.5% reported never smoking, 62.2% never vaping, and 20.7% never drinking alcohol. Firearm-related concerns were common. More than two-thirds of participants (67.9%) believed firearm violence was an issue in their community. Nearly half (45.8%) were somewhat worried and 33.3% were very worried about becoming a victim of firearm violence. Overall, 56.1% reported knowing at least one person who had been injured or killed by a firearm; 40.1% knew one to three affected individuals and 16.0% knew more than three.

The overall prevalence of clinically significant depressive symptoms (PHQ-9 ≥ 10) was 37.6% (n = 178). For PHQ-9 Item 9, which assesses thoughts of death or self-harm, 116 participants (24.5%) reported these thoughts on several days, 48 (10.1%) on more than half the days, and 23 (4.9%) nearly every day during the preceding two weeks. Overall, 187 participants (39.5%) endorsed Item 9 at any frequency other than “Not at all.”

Nativity was not associated with clinically significant depressive symptoms. Participants born outside the United States had similar odds of depressive symptoms compared with U.S.-born participants in crude (OR, 0.75; 95% CI, 0.52–1.09) and adjusted analyses (aOR, 0.80; 95% CI, 0.52–1.24; BH-adjusted p = 0.412). Among foreign-born participants, duration of residence in the United States was also not associated with depressive symptoms. Compared with participants who had lived in the United States for >20 years, the adjusted odds ratios were 0.99 (95% CI, 0.40–2.54) for 11–20 years, 1.21 (95% CI, 0.47–3.24) for 6–10 years, and 2.61 (95% CI, 0.64–10.99) for 2–5 years; none of these associations was statistically significant after multiple-comparison correction (Table 2).

**Table 2:** Crude and adjusted associations with clinically significant depressive symptoms (PHQ-9 ≥ 10) among Ethiopian and Ethiopian-descendant adults in the Seattle metropolitan area.

Exposure	PHQ-9 ≥ 10, n/N (%)	Crude OR (95% CI)	Adjusted OR (95% CI)	BH-adjusted p-value
**Born outside the United States**				
No	88/215 (40.9)	Ref	Ref	—
Yes	87/254 (34.3)	0.75 (0.52–1.09)	0.80 (0.52–1.24)	0.412
**Self-rated physical health**				
Excellent	17/142 (12.0)	Ref	Ref	—
Average	113/266 (42.5)	5.43 (3.17–9.81)	4.69 (2.63–8.77)	<0.001
Poor/Somewhat poor	44/60 (73.3)	20.22 (9.66–44.81)	18.99 (8.32–46.01)	<0.001
**Any chronic medical condition**				
No	116/340 (34.1)	Ref	Ref	—
Yes	62/134 (46.3)	1.66 (1.11–2.50)	1.61 (1.01–2.56)	0.082
**Ever used recreational marijuana or other drugs**				
No	90/336 (26.8)	Ref	Ref	—
Yes	88/138 (63.8)	4.81 (3.17–7.38)	5.02 (3.12–8.20)	<0.001
**Firearm exposure in the past year**				
No	48/223 (21.5)	Ref	Ref	—
Yes	130/251 (51.8)	3.92 (2.63–5.91)	3.93 (2.50–6.28)	<0.001
**Years in the United States, foreign-born participants only**				
> 20 years	13/38 (34.2)	Ref	Ref	—
11–20 years	30/107 (28.0)	0.75 (0.34–1.68)	0.99 (0.40–2.54)	0.981
6–10 years	30/80 (37.5)	1.15 (0.52–2.64)	1.21 (0.47–3.24)	0.781
2–5 years	8/15 (53.3)	2.20 (0.65–7.63)	2.61 (0.64–10.99)	0.274
**Ever given birth, female participants only**				
No	22/55 (40.0)	Ref	Ref	—
Yes	70/183 (38.3)	0.93 (0.50–1.74)	1.41 (0.62–3.34)	0.416
**Number of live births, parous women only**			**p for trend = 0.203**	
1	25/88 (28.4)	Ref	Ref	—
2	35/77 (45.5)	2.10 (1.11–4.04)	1.48 (0.65–3.39)	0.416
≥3	10/16 (62.5)	4.20 (1.41–13.53)	2.31 (0.53–9.95)	0.416
**History of postpartum depression, parous women only**				
No	18/110 (16.4)	Ref	Ref	—
Yes	51/70 (72.9)	13.72 (6.76–29.25)	9.76 (4.31–23.51)	<0.001

**Notes:** OR = odds ratio; CI = confidence interval; BH = Benjamini-Hochberg correction. Adjusted models were fit separately for each exposure and included age, sex, marital status, educational attainment, employment status, and nativity, except when nativity or duration of residence in the United States was the exposure. Years-in-U.S. analyses were restricted to foreign-born participants, with >20 years as the reference category. “Poor/Somewhat poor” combines the “Poor” and “Somewhat poor” response categories; “Not sure” responses were excluded from self-rated physical-health models. Adjusted complete-case sample sizes were N = 455 for nativity, chronic medical conditions, recreational marijuana or other drug use, and firearm exposure; N = 451 for self-rated physical health; and N = 233 for years-in-U.S. analyses. Reproductive-health models were restricted to the relevant subgroup and adjusted for age, marital status, educational attainment, employment status, and nativity; sex was not included because these analyses were restricted to female participants. Adjusted reproductive-model sample sizes were N = 226 for history of childbirth and N = 173 for parity and postpartum depression. BH correction was applied across 9 primary exposure comparisons and separately across 4 reproductive-health comparisons. The parity trend test was evaluated separately and was not included in the reproductive-health BH correction family.

Self-rated physical health was strongly associated with depressive symptoms. Compared with participants reporting excellent physical health, those reporting average physical health had higher odds of depressive symptoms (aOR, 4.69; 95% CI, 2.63–8.77; BH-adjusted p < 0.001), as did those reporting poor or somewhat poor physical health (aOR, 18.99; 95% CI, 8.32–46.01; BH-adjusted p < 0.001). Having any listed chronic medical condition was associated with higher odds of depressive symptoms before multiple-comparison correction (aOR, 1.61; 95% CI, 1.01–2.56; p = 0.045), but the association did not remain statistically significant after Benjamini-Hochberg correction (BH-adjusted p = 0.082). Ever use of recreational marijuana or other drugs was associated with higher odds of depressive symptoms (aOR, 5.02; 95% CI, 3.12–8.20; BH-adjusted p < 0.001). Participants who reported personally knowing someone who had been injured or killed by a firearm in their community during the past year also had higher odds of depressive symptoms (aOR, 3.93; 95% CI, 2.50–6.28; BH-adjusted p < 0.001).

In reproductive-health analyses, history of childbirth was not associated with clinically significant depressive symptoms among female participants (aOR, 1.41; 95% CI, 0.62–3.34; BH-adjusted p = 0.416). Among parous women, the proportion screening positive increased from 28.4% among those with one live birth to 45.5% among those with two births and 62.5% among those with three or more births. Although parity was associated with depressive symptoms in crude analyses, these associations were attenuated after adjustment. Compared with women with one live birth, the aOR was 1.48 (95% CI, 0.65–3.39) for two births and 2.31 (95% CI, 0.53–9.95) for three or more births; both BH-adjusted p-values were 0.416. The adjusted trend across parity categories was not statistically significant (OR per category increase, 1.51; 95% CI, 0.80–2.83; p for trend = 0.203). In contrast, history of postpartum depression was strongly associated with current clinically significant depressive symptoms. Among parous women, 72.9% of those reporting a history of postpartum depression had PHQ-9 scores ≥10 compared with 16.4% of those without such a history. The association remained strong after adjustment (aOR, 9.76; 95% CI, 4.31–23.51; BH-adjusted p < 0.001)

In the comprehensive mutually adjusted model, self-rated physical health, recreational marijuana or other drug use, and firearm exposure remained associated with clinically significant depressive symptoms (Table 3).

**Table 3:** Comprehensive mutually adjusted associations with clinically significant depressive symptoms (N = 451).

Exposure	Comparison	aOR (95% CI)	p-value	BH-adjusted p-value
Self-rated physical health	Average vs Excellent	3.83 (2.08–7.36)	<0.001	<0.001
	Poor/Somewhat poor vs Excellent	10.73 (4.53–26.80)	<0.001	<0.001
Any chronic medical condition	Yes vs No	1.21 (0.71–2.06)	0.482	0.482
Ever used recreational marijuana or other drugs	Yes vs No	3.52 (2.08–6.03)	<0.001	<0.001
Firearm exposure in the past year	Yes vs No	2.32 (1.39–3.90)	0.001	0.002
Born outside the United States	Yes vs No	0.74 (0.45–1.23)	0.242	0.291

**Notes:** The comprehensive logistic regression model included all listed exposures simultaneously and adjusted for age, sex, marital status, educational attainment, and employment status. Analysis was restricted to complete cases (N = 451). Benjamini-Hochberg correction was applied across the 6 primary exposure coefficients shown in the table.

Compared with participants reporting excellent physical health, those reporting average health had higher odds of depressive symptoms (aOR, 3.83; 95% CI, 2.08–7.36; BH-adjusted p < 0.001), while those reporting poor or somewhat poor health had substantially higher odds (aOR, 10.73; 95% CI, 4.53–26.80; BH-adjusted p < 0.001). Ever use of recreational marijuana or other drugs remained associated with higher odds of depressive symptoms (aOR, 3.52; 95% CI, 2.08–6.03; BH-adjusted p < 0.001), as did firearm exposure in the past year (aOR, 2.32; 95% CI, 1.39–3.90; BH-adjusted p = 0.002). Any chronic medical condition (aOR, 1.21; 95% CI, 0.71–2.06; BH-adjusted p = 0.482) and foreign-born status (aOR, 0.74; 95% CI, 0.45–1.23; BH-adjusted p = 0.291) were not associated with depressive symptoms after mutual adjustment.

Sensitivity analyses using PHQ-9 score as a continuous outcome were generally consistent with the primary findings. In the comprehensive linear regression model, poorer self-rated physical health, ever use of recreational marijuana or other drugs, and firearm exposure remained associated with higher PHQ-9 scores after mutual adjustment. Any chronic medical condition was not associated with PHQ-9 score in the comprehensive model. Foreign-born participants had modestly lower PHQ-9 scores after mutual adjustment (β=−1.02; 95% CI, −1.92 to −0.11; BH-adjusted p = 0.033) (Supplementary Tables S1 Table and S2 Table).

## Discussion

To our knowledge, this is among the first studies to characterize clinically significant depressive symptoms and associated factors among Ethiopian and Ethiopian-descendant adults in the Seattle metropolitan area. More than one-third of participants screened positive for clinically significant depressive symptoms using a PHQ-9 threshold of ≥10. In exposure-specific analyses, poorer self-rated physical health, ever use of recreational marijuana or other drugs, and firearm exposure in the past year were associated with higher odds of depressive symptoms. These associations remained statistically significant in the comprehensive model that simultaneously accounted for the primary exposures and sociodemographic covariates. Among parous women, a history of postpartum depression was also associated with current depressive symptoms, whereas childbirth and parity were not associated after adjustment.

The proportion of participants screening positive for clinically significant depressive symptoms was higher than estimates reported for the general U.S. adult population and several immigrant populations [27–33]. These comparisons should be interpreted cautiously because studies differ in sampling approaches, populations, measurement instruments, and time periods. In addition, participants in our study were recruited using nonprobability community-based sampling rather than population-based probability sampling. The 37.6% estimate reflects symptom burden in the study sample rather than the population prevalence among all Ethiopian or Ethiopian-descendant adults in the Seattle metropolitan area. Prior literature suggests that African immigrant communities may face barriers to mental health care, including stigma, language barriers, limited availability of culturally responsive services, and differences in conceptualizations of mental health and help-seeking [34,35]. These contextual factors may be relevant to understanding the substantial symptom burden observed in this sample.

Self-rated physical health showed one of the strongest associations with clinically significant depressive symptoms. In the comprehensive model, participants reporting average physical health had approximately four times the odds of depressive symptoms compared with those reporting excellent health, while those reporting poor or somewhat poor health had more than ten times the odds. Similar patterns were observed when the PHQ-9 score was analyzed continuously. These findings are consistent with prior literature documenting associations between poorer physical health, chronic disease burden, functional limitations, and depressive symptoms [34,35]. Poor physical health may contribute to psychological distress through functional limitations, pain, or reduced quality of life, while depressive symptoms may also influence perceptions of physical health. Since both were measured concurrently, the present study cannot determine the direction of this association. Because both self-rated physical health and depressive symptoms were self-reported, part of the observed association may also reflect shared method variance or a general tendency toward more negative self-appraisal among participants experiencing depressive symptoms. The attenuation of the association for any chronic medical condition in the comprehensive model further suggests that subjective physical-health status may capture aspects of health burden not fully reflected by the presence of diagnosed chronic conditions alone [36].

Ever use of recreational marijuana or other drugs was associated with higher odds of clinically significant depressive symptoms, consistent with epidemiologic literature documenting substantial co-occurrence between substance use and mood symptoms [37–40]. However, the cross-sectional design cannot establish whether recreational drug use preceded depressive symptoms, occurred in response to psychological distress, or reflected shared underlying factors. Future longitudinal studies that distinguish specific substances, frequency, recency, and patterns of use are needed to clarify this relationship.

Firearm-related exposure was also associated with depressive symptoms after mutual adjustment. Participants who reported knowing someone injured or killed by a firearm in their community during the past year had more than twice the odds of clinically significant depressive symptoms in the comprehensive model, with a similar pattern when the PHQ-9 score was modeled continuously. These findings are consistent with prior research documenting psychological distress, post-traumatic symptoms, grief, and depressive symptoms among individuals indirectly exposed to firearm violence through family, friends, and community networks [41–45]. However, the survey did not capture relationship closeness, circumstances of the firearm event, or cumulative exposure, and does not establish that firearm exposure caused subsequent depressive symptoms.

Among parous women, a history of postpartum depression was strongly associated with current clinically significant depressive symptoms. This finding is consistent with prior evidence that postpartum depression may identify women with greater vulnerability to subsequent depressive episodes [40,46,47]. Because postpartum depression history was retrospectively self-reported and current symptoms were assessed cross-sectionally, this association should be interpreted as a marker of prior mental-health vulnerability rather than evidence of a causal pathway.

The continuous PHQ-9 sensitivity analyses were generally consistent with the primary findings. Poorer self-rated physical health, recreational marijuana or other drug use, and firearm exposure remained associated with higher PHQ-9 scores in the comprehensive linear model. Foreign-born participants had modestly lower continuous PHQ-9 scores after mutual adjustment, despite no association with the PHQ-9 ≥ 10 threshold in the primary logistic analysis. This difference suggests that nativity may relate differently to symptom severity across the full PHQ-9 distribution than to crossing a specific screening threshold and should be interpreted cautiously.

This study has several strengths. It examines depressive symptoms in an Ethiopian and Ethiopian-descendant community that remains underrepresented in U.S. mental-health research. Recruitment through multiple community channels and the anonymous survey format may have facilitated participation and disclosure of sensitive information. Analytically, the study used prespecified covariate adjustment, reported exposure-outcome cell counts, applied Benjamini-Hochberg correction for multiple comparisons, fit a comprehensive model that simultaneously included the primary exposures, and evaluated robustness using continuous PHQ-9 scores. These approaches improve transparency and reduce reliance on a single model specification. The findings may have implications for community-based mental-health outreach. The high proportion of participants screening positive for clinically significant depressive symptoms, together with observed associations with poorer self-rated physical health, recreational marijuana or other drug use, firearm-related exposure, and prior postpartum depression, suggests that future mental-health assessment and outreach efforts may benefit from considering these broader health and social experiences.

## Limitations

This study should be interpreted in light of several limitations. First, the cross-sectional design prevents determination of temporal sequence and precludes causal inference. Associations involving physical health, recreational marijuana or other drug use, firearm exposure, and other factors may reflect reverse causation or bidirectional relationships. Second, the study used nonprobability convenience sampling through social media, community networks, churches, and community-based organizations. Individuals who were more socially connected, digitally engaged, comfortable participating in research, or interested in mental health may have been more likely to participate. Conversely, individuals who were socially isolated, had limited internet access, were less connected to community organizations, or were experiencing more severe mental-health or socioeconomic difficulties may have been underrepresented. These mechanisms could bias estimates in different directions, and their net effect cannot be determined. Third, the survey was administered only in English. This may have disproportionately excluded individuals with limited English proficiency, including some recent immigrants and older adults, and may have selected participants with greater English proficiency or U.S. acculturation. Fourth, all measures were self-reported and may be affected by recall error, social desirability bias, and misclassification. The PHQ-9 is a screening instrument rather than a clinical diagnostic assessment, and a score ≥10 identifies clinically significant depressive symptoms but does not establish major depressive disorder. Although the PHQ-9, including an Amharic-language version, has demonstrated validity among adults in Ethiopia, its measurement properties have not been specifically evaluated among Ethiopian and Ethiopian-descendant adults living in the United States [48,49]. Differences in language, migration context, and culturally specific expressions of distress may therefore influence symptom interpretation and endorsement. Fifth, some exposures were measured broadly. The recreational marijuana or other drug-use measure captured ever-use without distinguishing specific substances, frequency, recency, or severity. Similarly, the firearm-exposure item identified whether participants knew someone injured or killed by a firearm during the prior year but did not assess relationship closeness, circumstances of the event, or cumulative exposure. These measurement limitations constrain interpretation of the observed associations. Finally, although the analyses adjusted for prespecified sociodemographic covariates and included a comprehensive model that simultaneously accounted for the major exposures, residual confounding remains possible. Complete-case sample sizes varied across models, and subgroup analyses, particularly reproductive-health analyses, yielded some relatively imprecise estimates. Although Benjamini-Hochberg correction was used to address multiple comparisons, the findings should still be interpreted as exploratory and replicated in larger studies.

## Conclusion

Clinically significant depressive symptoms were common among Ethiopian and Ethiopian-descendant adults participating in this community-based study in the Seattle metropolitan area. Poorer self-rated physical health, ever use of recreational marijuana or other drugs, firearm exposure in the past year, and prior postpartum depression were associated with depressive symptom burden. These findings support the importance of culturally responsive mental-health screening and community-based research that considers physical health, behavioral health, reproductive mental-health history, and exposure to community violence. Larger probability-based and longitudinal studies, including surveys available in relevant Ethiopian languages, are needed to estimate population prevalence more accurately, establish temporal relationships, examine culturally specific manifestations of distress, and identify potentially modifiable factors that could inform prevention and treatment strategies.

## Supporting information

S1 Table Sensitivity analysis using continuous PHQ-9 score as the outcome. (DOCX)

S2 Table Comprehensive linear regression of mutually adjusted associations with continuous PHQ-9 score (N = 451). (DOCX)
