# Supplementary material for: Prevalence and correlates of depressive symptoms among Ethiopian and Ethiopian-Descent adults in the Seattle metropolitan area: A cross-sectional study
Source: PLOS Ment Health. 2026 Oct 21;3(10):e0000736. doi: 10.1371/journal.pmen.0000736 (PMC13686459; doi:10.1371/journal.pmen.0000736)
Supplement: Supplementary file 1 [file pmen.0000736.s001.docx]

S1 Table: Sensitivity analysis using continuous PHQ-9 score as the outcome

| Exposure | Comparison | Crude β (95% CI) | Crude p-value | Adjusted β (95% CI) | Adjusted P-value | BH-adjusted P-value |
| --- | --- | --- | --- | --- | --- | --- |
| Born outside the U.S. | Yes vs No | −1.24 (−2.31 to −0.17) | 0.024 | −0.83 (−1.89 to 0.24) | 0.127 | 0.247 |
| Self-rated physical health | Average vs Excellent | 4.28 (3.20 to 5.36) | <0.001 | 3.71 (2.65 to 4.78) | <0.001 | <0.001 |
|  | Poor/Somewhat poor vs Excellent | 8.26 (6.67 to 9.86) | <0.001 | 7.55 (5.94 to 9.16) | <0.001 | <0.001 |
| Any chronic medical condition | Yes vs No | 1.93 (0.76 to 3.10) | 0.001 | 1.66 (0.51 to 2.80) | 0.005 | 0.005 |
| Ever used recreational marijuana or other drugs | Yes vs No | 5.22 (4.15 to 6.30) | <0.001 | 4.63 (3.58 to 5.68) | <0.001 | <0.001 |
| Firearm exposure in past year | Yes vs No | 4.08 (3.08 to 5.09) | <0.001 | 3.56 (2.56 to 4.56) | <0.001 | <0.001 |
| Years in U.S., foreign-born only | 11–20 vs >20 | −1.85 (−3.98 to 0.28) | 0.089 | −1.31 (−3.56 to 0.94) | 0.251 | 0.228 |
|  | 6–10 vs >20 | 0.71 (−1.52 to 2.93) | 0.531 | 0.45 (−1.93 to 2.83) | 0.708 | 0.937 |
|  | 2–5 vs >20 | 0.93 (−2.51 to 4.37) | 0.596 | 0.52 (−3.11 to 4.14) | 0.779 | 0.937 |

**Notes:** β represents the mean difference in PHQ-9 score associated with each exposure. Adjusted models were fit separately for each exposure and included age, sex, marital status, educational attainment, and employment status. Nativity was additionally included except when nativity or years in the United States was the exposure of interest. Years-in-the-United-States analyses were restricted to foreign-born participants (N=240), with >20 years as the reference category. BH = Benjamini-Hochberg correction for multiple comparisons.
