# Supplementary material for: Prevalence and correlates of depressive symptoms among Ethiopian and Ethiopian-Descent adults in the Seattle metropolitan area: A cross-sectional study
Source: PLOS Ment Health. 2026 Oct 21;3(10):e0000736. doi: 10.1371/journal.pmen.0000736 (PMC13686459; doi:10.1371/journal.pmen.0000736)
Supplement: Supplementary file 2 [file pmen.0000736.s002.docx]

S2 Table: Comprehensive linear regression of mutually adjusted associations with continuous PHQ-9 score (N=451)

| Variable | Comparison | Adjusted β (95% CI) | P-value | BH-adjusted P-value |
| --- | --- | --- | --- | --- |
| Self-rated physical health | Average vs Excellent | 2.99 (1.98 to 3.99) | <0.001 | <0.001 |
|  | Poor/Somewhat poor vs Excellent | 5.73 (4.17 to 7.29) | <0.001 | <0.001 |
| Any chronic medical condition | Yes vs No | 0.77 (−0.22 to 1.77) | 0.127 | 0.127 |
| Ever used recreational marijuana or other drugs | Yes vs No | 3.37 (2.36 to 4.38) | <0.001 | <0.001 |
| Firearm exposure in the past year | Yes vs No | 1.81 (0.88 to 2.75) | <0.001 | <0.001 |
| Born outside the United States | Yes vs No | −1.02 (−1.92 to −0.11) | 0.028 | 0.033 |

**Notes:** The model simultaneously included self-rated physical health, any chronic medical condition, ever use of recreational marijuana or other drugs, firearm exposure, and nativity, and adjusted for age, sex, marital status, educational attainment, and employment status. β represents the adjusted mean difference in PHQ-9 score. Benjamini-Hochberg correction was applied to the exposure coefficients shown. Analysis was restricted to complete cases (N=451).
